# Pulmonary inflammation-induced loss and subsequent recovery of skeletal muscle mass require functional poly-ubiquitin conjugation

**DOI:** 10.1186/s12931-018-0753-8

**Published:** 2018-05-02

**Authors:** Judith J. M. Ceelen, Annemie M. W. J. Schols, Nathalie G. M. Thielen, Astrid Haegens, Douglas A. Gray, Marco C. J. M. Kelders, Chiel C. de Theije, Ramon C. J. Langen

**Affiliations:** 1 Department of Respiratory Medicine, School of Nutrition and Translational Research in Metabolism (NUTRIM), Maastricht University Medical Centre (MUMC+), Maastricht, the Netherlands; 2 Department of Biochemistry, Microbiology and Immunology, Ottawa Hospital Research Institute, Ottawa, Canada

**Keywords:** Inflammation, Skeletal muscle atrophy, Autophagy, Poly-ubiquitin, Proteolysis, Protein synthesis

## Abstract

**Background:**

Pulmonary inflammation in response to respiratory infections can evoke muscle wasting. Increased activity of the ubiquitin (Ub)-proteasome system (UPS) and the autophagy lysosome pathway (ALP) have been implicated in inflammation-induced muscle atrophy. Since poly-Ub conjugation is required for UPS-mediated proteolysis and has been implicated in the ALP, we assessed the effect of impaired ubiquitin conjugation on muscle atrophy and recovery following pulmonary inflammation, and compared activation and suppression of these proteolytic systems to protein synthesis regulation.

**Methods:**

Pulmonary inflammation was induced in mice by an intratracheal instillation of LPS. Proteolysis (UPS and ALP) and synthesis signaling were examined in gastrocnemius muscle homogenates. Ub-conjugation-dependency of muscle atrophy and recovery was addressed using Ub-K48R (K48R) mice with attenuated poly-ubiquitin conjugation, and compared to UBWT control mice.

**Results:**

Pulmonary inflammation caused a decrease in skeletal muscle mass which was accompanied by a rapid increase in expression of UPS and ALP constituents and reduction in protein synthesis signaling acutely after LPS. Muscle atrophy was attenuated in K48R mice, while ALP and protein synthesis signaling were not affected. Muscle mass recovery starting 72 h post LPS, correlated with reduced expression of UPS and ALP constituents and restoration of protein synthesis signaling. K48R mice however displayed impaired recovery of muscle mass.

**Conclusion:**

Pulmonary inflammation-induced muscle atrophy is in part attributable to UPS-mediated proteolysis, as activation of ALP- and suppression of protein synthesis signaling occur independently of poly-Ub conjugation during muscle atrophy. Recovery of muscle mass following pulmonary inflammation involves inverse regulation of proteolysis and protein synthesis signaling, and requires a functional poly-Ub conjugation.

**Electronic supplementary material:**

The online version of this article (10.1186/s12931-018-0753-8) contains supplementary material, which is available to authorized users.

## Background

Pulmonary inflammation may develop in response to respiratory infections or acute lung injury, and result in intensive care unit hospitalization and ICU-acquired muscle wasting [1]. Moreover, pulmonary inflammation often accompanies chronic obstructive pulmonary disease (COPD) exacerbations [2, 3]. Severe disease exacerbations in COPD often require hospital admission, and have been suggested to contribute to muscle wasting [4]. Muscle wasting contributes to a reduced quality of life and increased disability and mortality in COPD [5], and during or following critical illness [6]. Previous studies have shown that pulmonary inflammation is sufficient to induce muscle atrophy [7, 8], emphasizing the relevance of an improved understanding of the underlying mechanisms of loss and recovery of skeletal muscle mass to clinical conditions accompanied by acute pulmonary inflammation.

Protein turnover is an important determinant of muscle mass, and in homeostasis protein synthesis and breakdown rates are in balance. Muscle proteolysis involves multiple systems, including the ubiquitin 26S-proteasome system (UPS) and the autophagy lysosomal pathway (ALP) [9–11]. Proteasomal degradation of protein substrates requires conjugation of poly-ubiquitin chains. Although Ub conjugation can occur on various lysine residues, Ub K48-linkage is implicated as the main post-translational modification involved in Ub-mediated targeting of protein substrates to the 26S proteasome [12]. Ubiquitin conjugation is catalyzed by E3-ligases. In skeletal muscle, these include MuRF1 and Atrogin-1, and E3-ligase expression levels appear a rate limiting step in UPS-mediated proteolysis [13, 14]. The UPS and ALP have long been regarded as independent degradation pathways [15]. However, UPS dependency of autophagy termination [16], suggests that ALP mediated proteolysis may involve Ub conjugation.

Several studies have suggested UPS involvement in lung disease-associated muscle atrophy. Induction of E3 Ub-ligase expression in muscle following pulmonary inflammation has been documented [8], and Files et al. [7] have shown that muscle atrophy requires MuRF1 expression. However, as the expression of multiple E3 Ub ligases is concomitantly elevated during rapid muscle atrophy [17], the overall contribution of the UPS remains to be determined in pulmonary inflammation-induced muscle atrophy. Moreover, the role of ALP activation and suppression of protein synthesis signaling in inflammation-induced muscle wasting has received little attention, and their dependency on poly-ubiquitination has not been addressed. Finally, recovery of muscle mass following muscle atrophy involves a net increase in protein synthesis compared to proteolysis [18]. Although the dynamics in muscle mass have been described [8], UPS-, ALP-, and protein synthesis signaling during muscle mass recovery following pulmonary inflammation have not been explored.

We hypothesized that poly-ubiquitination is required for acute pulmonary inflammation-induced muscle atrophy, and that UPS-and ALP-related proteolysis signaling correlate inversely with protein synthesis signaling during muscle atrophy and recovery following pulmonary inflammation. To this end, muscle mass, UPS, ALP, and protein synthesis signaling in skeletal muscle were assessed following induction of pulmonary inflammation in transgenic mice expressing wild type ubiquitin (UBWT, control) or K48R-mutated ubiquitin, which impairs poly-ubiquitin conjugation.

## Materials and methods

### Animals and experimental protocol

All mouse studies were approved by the institutional Animal Care Committee of Maastricht University and the care and handling of the animals were in accordance with National Institutes of Health guidelines. Twelve-week-old male transgenic mice expressing a conjugation-terminating mutant form of Ub (K48R) and WT Ub expressing transgenic mice (UBWT) as appropriate controls on a FVB background [19, 20], were allowed food and water ad libitum throughout experiments. Mice received intratracheal (IT) instillation of a bolus (50 μl) LPS solution (0.6 μg per gram mouse, Escheria coli, serotype o55:B5, Sigma, St. Louis, MO [8]) to induce lung inflammation or 50 μl sterile saline (vehicle control). Body weights and food intake were recorded throughout the experiment. With the exception of 7 h (UBWT mice only), UBWT and K48R mice were sacrificed 24, 48, 72, 96, and 120 h after LPS (*n* = 5–7/time-point) or saline (*n* = 3–4/time-point) instillation and gastrocnemius muscle was collected, weighed and stored in − 80 °C for further analysis. At 48 h after LPS, also lungs were collected for mRNA analysis of inflammation markers.

### Histological analysis

The lungs were fixated by infusion of 4% paraformaldehyde through a tracheal cannula and excised for quantitative assessment of lung structure [21, 22]. The lung lobes were embedded in paraffin, and sections were stained with haematoxylin and eosin staining to confirm pulmonary inflammation.

### RNA isolation

Total RNA was isolated from homogenized gastrocnemius muscle using the TRI REAGENT™ (Sigma-Aldrich Chemie B.V, Zwijndrecht, NL). Before precipitation with isopropanol, glycogen (Invitrogen 10,814–010) was added as co-precipitant according to the manufacturer’s instructions. cDNA was made with the Tetro cDNA Synthesis kit (GC biotech). qPCR primers were designed using Primer Express 2.0 software (applied Biosystems) and ordered from Sigma Genosys (Table 1). The relative DNA starting quantities of the samples were derived using LinRegPCR software (Version 2014.0, Ruijter). The expression of genes of interest was normalized to the geometric average of three or four reference genes (cyclophilin A, beta-2-microglobulin, GAPDH, RPLP0, GUSB) by the GeNorm software.

**Table 1:** Sequences of primers used for RT-qPCR to assess expression of the indicated genes

Gene	Forward primer (5′ to 3′)	Reverse primer (5′ to 3′)
Cyclophilin A	TTCCTCCTTTCACAGAATTATTCCA	CCGCCAGTGCCATTATGG
Beta-2-microglobulin	CTTTCTGGTGCTTGTCTCACTGA	GTATGTTCGGCTTCCCATTCTC
GAPDH	CAACTCACTCAAGATTGTCAGCAA	TGGCAGTGATGGCATGGA
RPLP0	GGACCCGAGAAGACCTCCTT	GCACATCACTCAGAATTTCAATGG
GUSB	CATTAGCAAGCTGGTCCAGAGT	GACAAAGTAACCCTTGGGATACAT
MuRF1	CTTCCTCTCAAGTGCCAAGCA	GTGTTCTAAGTCCAGAGTAAAGTAGTCCAT
Atrogin-1	CAGCAGCTGAATAGCATCCAGAT	TCTGCATGATGTTCAGTTGTAAGC
LC3B	GAGCAGCACCCCACCAAGAT	CGTGGTCAGGCACCAGGAA
p62/SQSTM1	GAATGTGGGGGAGAGTGTGG	TCTTCTGTGCCTGTGCTGGA
REDD1	TCGGCGCTTCACTACTGACC	CCTAACACCCACCCCATTCC
FoXO1	AAGAGCGTGCCCTACTTCAAGGATA	CCATGGACGCAGCTCTTCTC
IL-6	GTATGAACAACGATGATGCACTTG	GAAGACCAGAGGAAATTTTCAATAGG
TNF-α	CAGCGCTGAGGTCAATCTGCC	TGCCCGGACTCCGCAA
CXCL1	TCGTCTTTCATATTGTATGGTCAACACG	TGCCCTACCAACTAGACACAAAATGTC

### Western blotting

Gastrocnemius muscle was ground to powder using an N_2_-cooled steel mortar. The powder (~ 20 mg) was lysed in 600 μl lysis buffer [50 mM Tris, pH 7.4; 150 mM NaCl; 10% glycerol; 0,05% Nonidet P-40; 1 mM EDTA; 500 μM Na3VO4; 500 μM NaF, 100 μM β-glycerophosphate; 100 μM sodium pyrophosphate; 1 mM DTT, 10 μg/mL Leupeptin and 1% Aprotenin] (all chemicals from Sigma-Aldrich Chemie, Zwijndrecht, Netherlands), and protease inhibitors (Complete; Roche Nederland, Woerden, Netherlands), using a mini-BeadBeater. Lysates were incubated at 4 °C in a tube rotator for 60 min, followed by 30-min centrifugation at 14,000 *g*. Pellet fractions were stored at − 80 °C for future analysis. Total protein concentration of the supernatant was determined with a BCA protein assay kit (Pierce Biotechnology, #23225, Rockford, IL) according to manufacturer’s instructions. To part of the supernatant fraction 4× laemmli buffer [0.25 M Tris, pH 6.8; 8% SDS; 40% glycerol; 0.4 M DTT and 0.02% Bromophenol Blue] was added and denatured by heating at 100 °C for 5 min. Samples were analyzed by western blot. Briefly, 10 μg of protein per lane were separated on a CriterionTM XT Precast 4–12% or 12% Bis-Tris gel (Bio-Rad Laboratories, Veenendaal, Netherlands) and transferred to a nitrocellulose transfer membrane (Bio-Rad Laboratories) by electroblotting. The membrane was stained with Ponceau *S* solution (0.2% Ponceau S in 1% acetic acid; Sigma-Aldrich Chemie) to control for equal protein loading. The membrane was blocked for 1 h at room temperature in 3% (wt/vol) nonfat dried milk (Campina, Zaltbommel, Netherlands) dissolved in TBS-Tween-20 (0.05%). Nitrocellulose blots were washed in TBS-Tween-20 (0.05%) on a rocking platform for 5 min, followed by overnight incubation at 4 °C with primary antibodies [AKT: no. 9272; p-AKT(Ser473): no. 9271; FOXO1: no. 2880; p-FOXO1(Ser256): no. 9461; TSC2: no. 4308; p-TSC2(Thr1462): no. 3617; mTOR (7C10): no. 2983; p-mTOR(Ser2448): no. 2971; S6: no. 2217; p-S6(Ser235/236): no. 4856; P70S6K1: no. 9202; p-P70S6K1(Thr389): no. 9205; 4EBP1: no. 9452; p-4EBP1(Thr37/46): no. 9459; p-4EBP1(S65): no. 9451; ULK1: no. 8054; p-ULK1(Ser757): no. 6888; LC3B: no. 2775; Sqstm1/p62: no. 5114 (Cell Signaling Technology, Beverly, MA) and REDD1: no. 10638–1-AP (ProteinTech, Manchester, UK)]. All antisera were diluted 1/1000 in TBS-Tween-20 (0.05%). After three washing steps of 10 min each, blots were probed with a horseradish peroxidase-conjugated secondary antibody (Vector Laboratories, Burlingame, CA) and visualized with chemiluminescence (Supersignal West Pico or Femto Chemiluminescent Substrate; Pierce Biotechnology) in a LAS-3000 Luminescent Image analyzer (Fujifilm, Tokyo, Japan). Bands were quantified using the Quantity One software (Bio-Rad, version 4.5.0). All data were corrected for equal protein loading as determined after Ponceau S staining.

### Statistical analyses

Data are shown as means ± SE. Comparisons were computed using SPSS version 22.0. For assessment of significance between groups and genotypes an independent samples T-test was used. Interactions between genotypes and treatment were assessed using a two-way ANOVA. A *p-*value < 0.05 was considered statistically significant.

## Results

### Pulmonary inflammation-induced muscle atrophy and subsequent muscle mass recovery require poly-ubiquitin conjugation

**Fig. 1:**
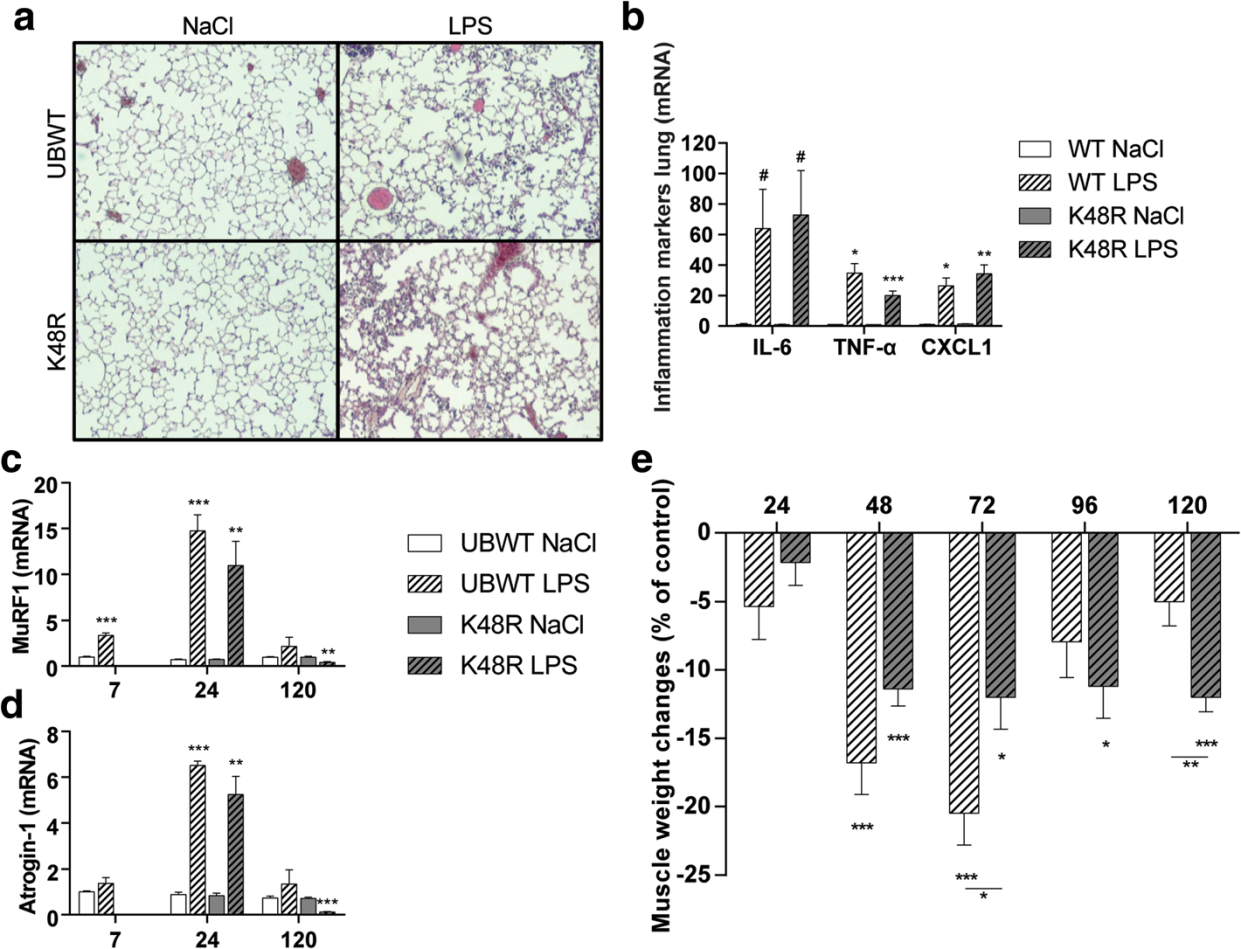
*Pulmonary inflammation-induced muscle atrophy and subsequent muscle mass recovery require poly-ubiquitin conjugation*. UBWT and K48R mice were instilled intratracheally with NaCl or LPS. At 48 h after LPS **a)** lung sections were stained with H&E to confirm pulmonary inflammation, or **(B)** mRNA levels of inflammatory markers IL-6, TNFα and CXCL1 were determined in lung tissue from WT and K48R mice. At 7 (UBWT only), 24 and 120 h after LPS, mRNA abundance of **(c)** MuRF1 and **(d)** Atrogin-1 were assessed in gastrocnemius muscle tissue. **e)** Gastrocnemius wet weights were measured and expressed as a percentage of their respective IT-NaCl time control to represent the response to pulmonary inflammation. All data shown represent means ±SEM. * *p* < 0.05, ** *p* < 0.01, *** *p* < 0.001 compared with control (intratracheal NaCl), * above a line refers to a difference in response between genotypes. # represents a trend

As expected, IT-instillation of LPS evoked pulmonary inflammation involving inflammatory cell recruitment (Fig. 1A) and increased expression of pro-inflammatory cytokines and chemokines (Fig. 1B), was similar in control and K48R transgenic mice. In addition, alterations in body weight and food-intake following pulmonary inflammation did not differ between the genotypes (Additional file 1A-B). Increased mRNA transcript levels of the muscle specific E3 ubiquitin ligases Atrogin-1 and MuRF1 24 h after LPS instillation (Fig. 1C, D) confirmed intact activation of upstream UPS signaling in UBWT and K48R mice. After LPS, a rapid decrease in skeletal muscle mass was observed which again recovered as of 72 h (Fig. 1E). Notably, loss of muscle mass was less pronounced in K48R mice, but not completely prevented, suggesting additional involvement of processes that determine protein turnover and muscle mass independently of poly-Ub conjugation. Furthermore, muscle mass from the K48R mice did not recover over this timeframe, indicating that poly-Ub conjugation is essential for recovery of muscle mass after inflammation-induced atrophy.

### Transient activation of autophagy during muscle atrophy following pulmonary inflammation

**Fig. 2:**
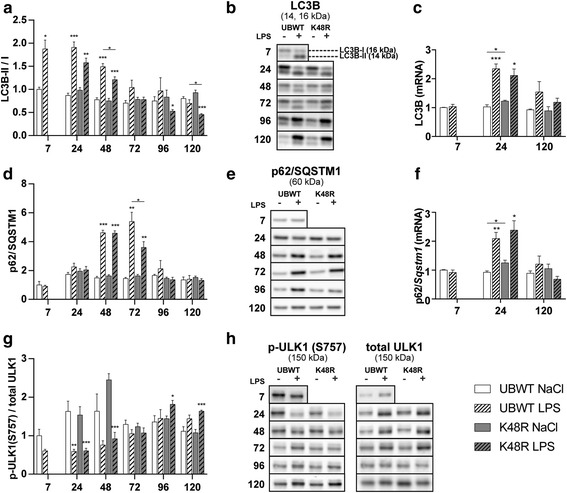
*Transient activation of autophagy during muscle atrophy following pulmonary inflammation*. UBWT and K48R mice were subjected to the intratracheal instillation of NaCl or LPS, and gastrocnemius muscle was collected at the indicated time points (hours after instillation). Protein levels of **(b)** LC3B-I and LC3B-II, **(d, e)** p62, and **(h)** phosphorylated ULK1 (ser757) and total ULK1 were assessed in lysates of gastrocnemius muscle tissue with western blot analysis. **a)** Ratio of LC3B-II over LC3B-I. **g)** Ratio of phosphorylated ULK1 over total ULK1. mRNA transcript levels of **(c)** LC3B and **(f)** p62 were determined, normalized to geNorm, and expressed as fold change compared with UBWT intratracheal NaCl. * *p* < 0.05, ** *p* < 0.01, *** *p* < 0.001 compared with control (intratracheal NaCl), * above a line refers to a difference in response between genotypes

During active autophagy the cytosolic form of LC3 (LC3B-I) is conjugated to the lipidated form (LC3B-II), resulting in recruitment to the autophagosomal membrane [23]. In both UBWT and K48R mouse muscle, the LC3B-II/LC3B-I ratio was significantly increased up to 48 h post LPS (Fig. 2A, B). This was accompanied by increased mRNA levels of LC3B after LPS (Fig. 2C), suggesting increased conversion of LC3B-I into LC3B-II, and an increased autophagic flux. As of 72 h post LPS, the LC3BI/II ratio returned to control levels. 120 h post LPS a further reduction was observed in K48R mice only, which resulted from increased (3-fold, *p* < 0.001) LC3B-I expression, indicating suppression of the ALP. p62 can facilitate the clearance of ubiquitinated proteins by targeting to the autophagosome [24]. p62 protein abundance was increased 48 and 72 h post LPS (Fig. 2D, E), and was preceded by increased p62 mRNA levels (Fig. 2F) in both UBWT and K48R mice. ULK1 signaling stimulates autophagosome formation, and is inhibited by mTOR via phosphorylation on ser757 [25, 26]. In both UBWT and K48R mouse muscle, ULK1 ser757 phosphorylation was significantly decreased 24 to 48 h post LPS (Fig. 2G, H), suggesting increased autophagy initiation and corresponding with the elevated LC3B ratio and p62 levels during acute loss of muscle mass. 120 h after LPS, ULK1 phosphorylation was increased, particularly in K48R muscle, in line with the suppressed LC3B-II/I ratio, suggesting inhibition of the ALP. Collectively in both genotypes, ALP markers suggested activation of autophagy preceding the maximally observed muscle atrophy. Autophagic activity in UBWT returned to baseline over time, whereas in K48R mice the ALP appeared further repressed compared to wildtype.

### Changes in protein synthesis signaling correspond to muscle mass loss and recovery following pulmonary inflammation

**Fig. 3:**
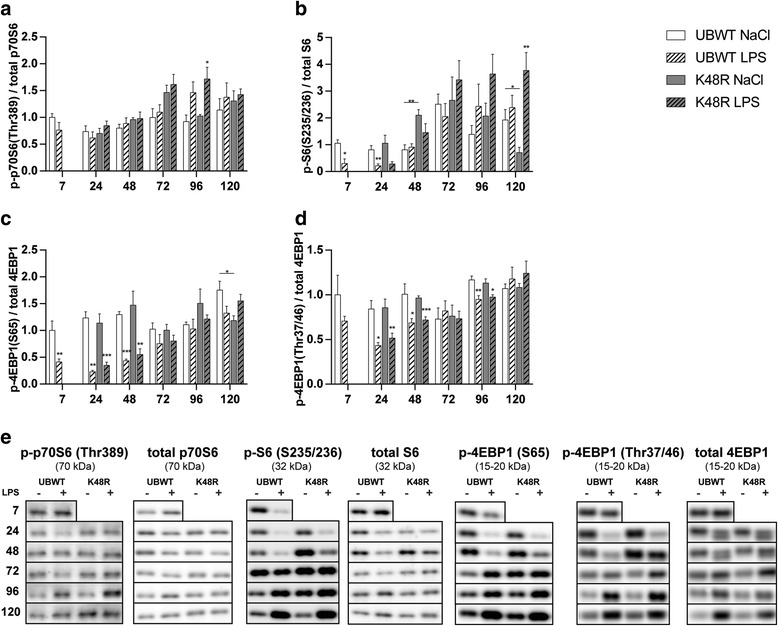
*Changes in protein synthesis signaling correspond to muscle mass loss and recovery following pulmonary inflammation. UBWT and K48R* mice were subjected to the intratracheal instillation of NaCl or LPS, and gastrocnemius muscle was collected at the indicated time points (hours after instillation). Protein levels of phosphorylated p70S6 (Thr389), total p70S6, phosphorylated S6 (ser235/236), total S6, phosphorylated 4EBP1 (ser65 and Thr37/46) and total 4EBP1 were assessed in lysates of gastrocnemius muscle tissue with western blot analysis. **(e)** Representative western blots of the indicated proteins. Ratios of **(a)** phosphorylated p70S6 over total p70S6, **(b)** phosphorylated S6 over total S6, **(c)** phosphorylated 4EBP1 (ser65) over total 4EBP1 and **(d)** phosphorylated 4EBP1 (Thr37/46) over total 4EBP1. * *p* < 0.05, ** p < 0.01, *** *p* < 0.001 compared with control (intratracheal NaCl), * above a line refers to a difference in response between genotypes

Next, the phosphorylation status of p70S6, S6 and 4EBP1, which control mRNA translation as rate-limiting step of protein synthesis, were determined (Fig. 3E). Although p70S6 phosphorylation did not change between 7 to 48 h after LPS (Fig. 3A), phosphorylation of its downstream substrate S6 was significantly decreased (Fig. 3B). 4EBP1 ser65 and Thr37/46 phosphorylation comparably decreased (Fig. 3C, D). Muscle mass recovery apparent after 72 h post LPS, was accompanied by restoration of p70S6 phosphorylation towards control levels (Fig. 3A). Phosphorylation of 4EBP1 (S65: (1.8- and 1.6-fold, T37/46 1.5- and 1.7-fold) was significantly increased 96 h post-LPS in UBWT and K48R mice, respectively, but this was masked in the phosphorylated/total ratio (Fig. 3C, D) by concomitant increases in total abundance of these proteins (Fig. 3E). Of note, S6 phosphorylation was markedly increased in the K48R mice at 120 h after LPS (Fig. 3E).

### Increased REDD1 expression accompanies inhibited protein synthesis signaling following pulmonary inflammation

**Fig. 4:**
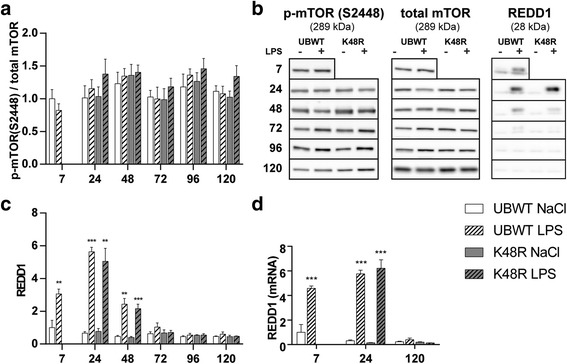
*Increased REDD1 expression accompanies inhibited protein synthesis signaling following pulmonary inflammation.* UBWT and K48R mice were subjected to the intratracheal instillation of NaCl or LPS, and gastrocnemius muscle was collected at the indicated time points (hours after instillation). Protein levels of phosphorylated mTOR (ser2448), total mTOR or **(c)** REDD1 were assessed in lysates of gastrocnemius muscle tissue with western blot analysis. **a)** Ratio of phosphorylated mTOR over total mTOR. **b)** Representative western blots of the indicated proteins. **d)** mRNA transcript levels of REDD1 were determined, normalized to geNorm, and expressed as fold change compared with UBWT intratracheal NaCl. *** *p* < 0.001 compared with control (intratracheal NaCl)

p70S6 and 4EBP1 are controlled by mTORC1 activity, which in turn is regulated by REDD1 [27]. In line with reduced mTORC1 activity, mRNA transcript (Fig. 4D) and protein (Fig. 4C) abundance of REDD1 was highly increased acutely after LPS instillation in both UBWT and K48R muscle, and returned to baseline at 120 h post LPS. mTOR is also regulated through Akt-mediated phosphorylation at ser2448 [28], but no changes were found after LPS instillation (Fig. 4A-B).

### Dynamic regulation of FoXO1 activity during muscle atrophy and recovery following pulmonary inflammation

**Fig. 5:**
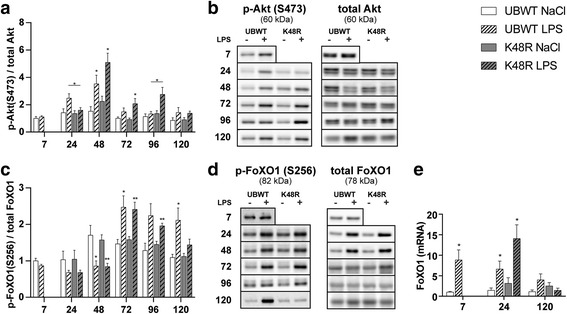
*Dynamic regulation of FoXO1 activity during muscle atrophy and recovery following pulmonary inflammation.* UBWT and K48R mice were subjected to the intratracheal instillation of NaCl or LPS, and gastrocnemius muscle was collected at the indicated time points (hours after instillation). Protein levels of phosphorylated Akt (ser473), total Akt, phosphorylated FoXO1 (ser256) and total FoXO1 were assessed in lysates of gastrocnemius muscle tissue with western blot analysis. **b, d)** Representative western blots of the indicated proteins. Ratios of **(a)** phosphorylated Akt over total Akt and **(c)** phosphorylated FoXO1 over total FoXO1. **e)** mRNA transcript levels of FoXO1 were determined, normalized to geNorm, and expressed as fold change compared with UBWT intratracheal NaCl. * *p* < 0.05, ** p < 0.01 compared with control (intratracheal NaCl), * above a line refers to a difference in response between genotypes

Akt is not only the upstream effector of mTOR, but also of FoXO1. Akt ser473 phosphorylation was increased 48 h post LPS (Fig. 5A-B). The expression of Atrogin-1 and MuRF1 is regulated by activation of FoXO family of transcription factors. Phosphorylation on ser256 by Akt results in nuclear export and inhibition of transcriptional activity of FoXO1 [29]. In both UBWT and K48R mouse muscle, the ratio of phosphorylated to total FoXO1 was significantly decreased 24 to 48 h post LPS (Fig. 5C). However, this was the result of strongly increased total FoXO1 levels (24 h: 3.1- and 2.5-fold, 48 h: 4- and 3.4-fold), in UBWT and K48R mice, respectively, which overwhelmed increases in abundance of phosphorylated FoXO1 (Fig. 5D). 72 h post LPS, total FoXO1 levels returned to baseline while phosphorylated levels remained increased. This corresponded with Akt phosphorylation and initiation of muscle mass recovery. FoXO1 mRNA levels were increased acutely after LPS, again returning to baseline after 120 h (fig. 5E). Combined, these data imply rapid de-repression of FoXO1 activity and subsequent increases in its expression in acute atrophying muscle, followed by Akt-mediated inhibitory phosphorylation of FoXO1 during muscle mass recovery.

## Discussion

Therapeutic strategies to prevent ICU-acquired or COPD exacerbation-associated muscle wasting are currently lacking, and restoration of lost muscle mass and function following recovery from critical illness or COPD exacerbation is challenging and often incomplete [30, 31]. Pulmonary inflammation often accompanies these conditions and is sufficient to induce muscle atrophy, but the intracellular mechanisms governing the net loss of muscle protein have not completely been identified. Although individual E3 ligases of the UPS have been implicated previously [7, 8], using a comprehensive approach employing K48R transgenic mice to inhibit poly-ubiquitin conjugation, we now demonstrate partial dependency of pulmonary inflammation-driven muscle atrophy on UPS-mediated proteolysis. The Ub K48R substitution interferes with assembly of ubiquitin chains with the topology required for proteasomal targeting [32], but not with upstream activation of the UPS proteolytic program. Accordingly, the induction of Atrogin-1 and MuRF1 expression is similar to UBWT mice. Moreover, as the Ub K48R substitution does not affect ALP- and protein synthesis signaling during muscle loss, we conclude that muscle atrophy observed following pulmonary inflammation is in part dependent on UPS-mediated proteolysis. This is in line with other studies demonstrating partial prevention of atrophy in limb muscle [33] and respiratory muscle [34], and demonstrates a similar reliance on UPS-mediated atrophy of muscles of varying fiber type composition. The residual muscle mass loss in K48R mice observed up to 72 h post-LPS implies a contribution of increased autophagy and reduced protein synthesis signaling to muscle atrophy following pulmonary inflammation.

The increased expression levels and post-translational alterations of proximal (LC3B, p62) and distal (ULK1) ALP constituents early after induction of pulmonary inflammation, correspond with rapidly developing loss of muscle mass, in line with other reports demonstrating activation of the autophagy machinery in acute muscle atrophy [35]. ULK1 is an upstream regulator of autophagy, and its activity corresponds inversely with its phosphorylation on serine 757 catalyzed by mTORC1 [25]. The rapid reduction in serine 757 ULK1 phosphorylation levels is accompanied by increases in LC3B-II/-I ratio, implying decreased mTORC1 activity in the initiation of autophagy. In line with this notion, other downstream targets of mTOR signaling, i.e. 4EBP1 and S6, also display reduced phosphorylation levels reflective of decreased mTORC1 activity in the initial phases of muscle atrophy. These findings correspond with studies showing that inhibition of mTOR is sufficient to initiate autophagy in skeletal muscle [25]. mTORC1 activity in skeletal muscle is subject to regulation by REDD1, which stimulates the inhibitory actions of TSC2 on mTORC1 [27]. As mRNA and protein expression levels of REDD1 are rapidly increased after induction of pulmonary inflammation, REDD1-mediated mTORC1 complex inhibition may represent the first step in activation of the ALP. In line with this notion, it has previously been shown that induction of autophagy in skeletal muscle in response to systemic inflammation requires REDD1 expression [36, 37]. Another important group of upstream regulators of autophagy are the FoXOs which are required to sustain autophagic flux by upregulating autophagy-related gene transcription like p62 and LC3 [38, 39]. The decreased phosphorylated over total FoXO1 protein abundance, suggesting increased FoXO activity, accompany elevated LC3B and p62 mRNA levels during muscle atrophy following pulmonary inflammation, in support of FoXO as a transcriptional regulator of these genes [39, 40]. Combined, these data suggest involvement of autophagy-mediated degradation in pulmonary inflammation-induced muscle atrophy through mTOR inhibition and FoXO1 activation, in addition to UPS-mediated proteolysis.

Others have postulated that muscle atrophy after acute inflammation is not only the result of increased proteolysis, but also of reduced protein synthesis [41–43]. In this study, levels of phosphorylated 4EBP1 and S6 decrease early after LPS, indicating reduced cap-dependent mRNA translation during muscle atrophy. This rate limiting step of protein synthesis is controlled by mTORC1 signaling [44]. As these changes are not accompanied by altered levels of phosphorylated mTOR (S2448) or TSC2 (T1462) (data not shown), this indicates that reduced mTORC1 activity suggested by the decreased p-4EBP1 and p-S6 levels, is not a consequence of alterations in Akt signaling [28, 45, 46]. Instead, the reduction of these proximal markers of protein synthesis more likely reflects REDD1-mediated inhibition of mTORC1, in line with previously reported inhibition of mTORC1 activity and protein synthesis in inflammation-induced atrophy [37]. Combined, these data suggest a contribution of reduced protein synthesis signaling in pulmonary inflammation-induced muscle atrophy in addition to UPS- and ALP-mediated proteolysis.

### UPS and ALP inversely correlate with protein synthesis signaling during loss and recovery of muscle mass

Indicative of coordinated activation of the UPS and the ALP following pulmonary inflammation, increased levels of the E3 ligases MuRF1 and Atrogin-1 correspond with increased levels of LC3B-II and decreased phosphorylation of ULK1. Moreover, protein synthesis signaling is decreased. This shift in protein turnover regulation in favor of proteolysis likely drives the observed maximal muscle mass decreases after 48 h. The increased p-FoXO1/total-FoXO1 ratio 72 h following induction of inflammation marks attenuation of the protein breakdown machinery. Indeed both transcripts encoding UPS (MuRF1, Atrogin-1) and ALP (LC3B, p62), which are under transcriptional control of FoXO [39, 40], return to baseline. Conversely, protein synthesis signaling restores or even increases 72 h after LPS. These dynamics in proteolysis and protein synthesis signaling represent a shift in favor of synthesis at the later time points, which corresponds with the recovery of muscle mass. This confirms the notion that UPS- and ALP-related proteolysis and protein synthesis signaling correlate inversely during muscle atrophy and muscle mass recovery following pulmonary inflammation.

### Ub conjugation is required for muscle mass recovery following pulmonary inflammation

While muscle atrophy is almost completely restored in the UBWT mice, no muscle mass recovery is observed in K48R mice within the timeframe assessed in this study. This is consistent with earlier findings that a functional UPS is necessary for skeletal muscle growth and remodeling [47], and regeneration [48, 49]. Whereas in UBWT mice ULK1 Ser757 phosphorylation and the ratio of LC3B-II/I return to baseline levels during muscle mass recovery, this is not observed in K48R mice. It has been previously shown that termination of autophagy is dependent on UPS-mediated turnover of ULK1 [16], and impaired termination of autophagy affects the amplitude and duration of muscle atrophy [16]. This suggests that impaired ALP may contribute to disturbed muscle mass recovery in K48R mice. Accordingly, inappropriate activation as well as inhibition of autophagy in skeletal muscle result in myopathy and muscle atrophy [50].

Conversely, levels of phosphorylated S6 remain upregulated in K48R mice, which may reflect a futile attempt of the protein synthesis machinery to compensate for the inability to regain muscle mass. Although the exact mechanism for the sustained muscle atrophy remains unclear, these findings suggest that disturbances of processes involved in protein turnover result in impaired muscle mass recovery following atrophy.

## Conclusions

In summary, this study reveals that muscle atrophy in response to pulmonary inflammation can be partitioned in UPS-mediated proteolysis, and a contribution of increased autophagy and reduced protein synthesis signaling, which provides leads for the development of future interventions on separate processes to modulate muscle wasting. As we also demonstrate that functional Ub conjugation is required for muscle mass recovery following pulmonary inflammation-induced muscle atrophy, this illustrates that the effects of candidate therapeutics should be evaluated on all aspects of muscle mass plasticity.

## Additional file

Additional file 1: Changes in food intake, body- and muscle weight following pulmonary inflammation. **A)** Food intake was recorded throughout the experiment. **B)** Body weights were measured and weight change per 24 h timeframes was expressed as a percentage. **C)** Gastrocnemius wet weights corrected for starting body weight and **(D)** the combined wet weights of the soleus, plantaris and gastrocnemius muscle (SPG complex) were measured and expressed as a percentage of their respective IT-NaCl time control to represent the response to pulmonary inflammation. All data shown represent means ±SEM. * *p* < 0.05, ** *p* < 0.01, *** *p* < 0.001 compared with control (intratracheal NaCl), * above a line refers to a difference in response between genotypes. (TIFF 9317 kb)

## Abbreviations

ALP: Autophagosomal-lysosomal pathway
COPD: Chronic Obstructive Pulmonary Disease
FVB: Friend Virus B
IT: Intratracheal instillation
Ub: ubiquitin
UPS: Ubiquitin proteasome system
WT: Wildtype
