# Supplementary figures and images for: Pulmonary inflammation-induced loss and subsequent recovery of skeletal muscle mass require functional poly-ubiquitin conjugation
Source: Respir Res. 2018 May 2;19:80. doi: 10.1186/s12931-018-0753-8 (PMC5932886; doi:10.1186/s12931-018-0753-8)

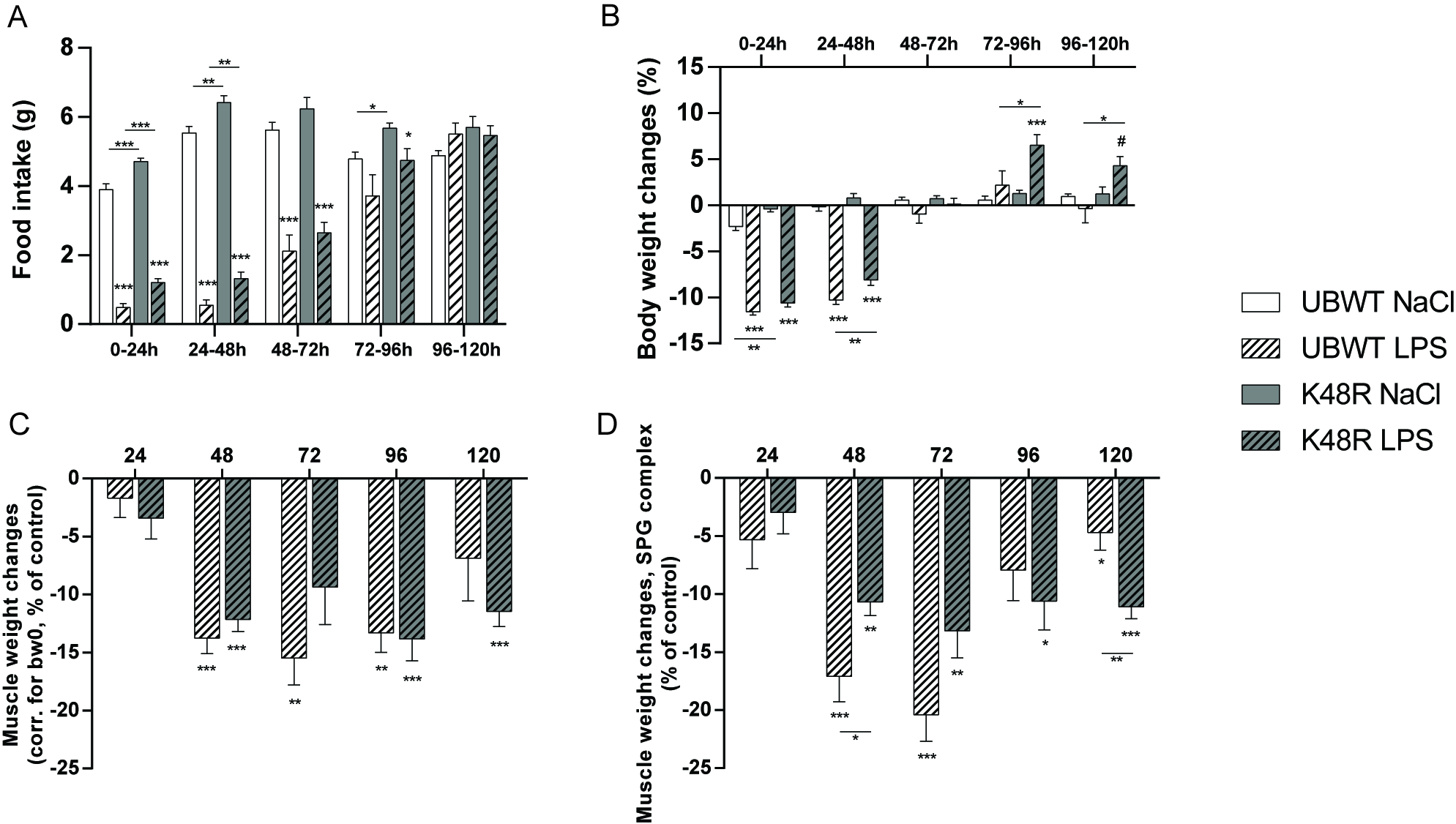

Supplement: Supplementary file 1 — Changes in food intake, body- and muscle weight following pulmonary inflammation. A) Food intake was recorded throughout the experiment. B) Body weights were measured and weight change per 24 h timeframes was expressed as a percentage. C) Gastrocnemius wet weights corrected for starting body weight and (D) the combined wet weights of the soleus, plantaris and gastrocnemius muscle (SPG complex) were measured and expressed as a percentage of their respective IT-NaCl time control to represent the response to pulmonary inflammation. All data shown represent means ±SEM. * p < 0.05, ** p < 0.01, *** p < 0.001 compared with control (intratracheal NaCl), * above a line refers to a difference in response between genotypes. (TIFF 9317 kb) [file 12931_2018_753_MOESM1_ESM.tif]
